# On homogeneous second order linear general quantum difference equations

**DOI:** 10.1186/s13660-017-1471-3

**Published:** 2017-08-24

**Authors:** Nashat Faried, Enas M Shehata, Rasha M El Zafarani

**Affiliations:** 1 Department of Mathematics, Faculty of Science, Ain Shams University, Cairo, Egypt; 2 Department of Mathematics, Faculty of Science, Menoufia University, Shibin El-koom, Egypt

**Keywords:** 39A10, 39A13, 39A70, 47B39, a general quantum difference operator, general quantum difference equations, Euler-Cauchy general quantum difference equation

## Abstract

In this paper, we prove the existence and uniqueness of solutions of the *β*-Cauchy problem of second order *β*-difference equations

$$a_{0}(t)D_{\beta}^{2}y(t)+a_{1}(t)D_{\beta}y(t)+a_{2}(t)y(t)=b(t),\quad t \in I, $$

$a_{0}(t)\neq0$, in a neighborhood of the unique fixed point $s_{0}$ of the strictly increasing continuous function *β*, defined on an interval $I\subseteq{\mathbb{R}}$. These equations are based on the general quantum difference operator $D_{\beta}$, which is defined by $D_{\beta}{f(t)}= (f(\beta(t))-f(t) )/ (\beta(t)-t )$, $\beta(t)\neq t$. We also construct a fundamental set of solutions for the second order linear homogeneous *β*-difference equations when the coefficients are constants and study the different cases of the roots of their characteristic equations. Finally, we drive the Euler-Cauchy *β*-difference equation.

## Introduction

Quantum calculus allows us to deal with sets of non-differentiable functions by substituting the classical derivative by a difference operator. Non-differentiable functions are used to describe many important physical phenomena. Quantum calculus has a lot of applications in different mathematical areas such as the calculus of variations, orthogonal polynomials, basic hyper-geometric functions, economical problems with a dynamic nature, quantum mechanics and the theory of scale relativity; see, *e.g.*, [1–9]. The general quantum difference operator $D_{\beta}$ is defined, in [10, p.6], by

$$ {D}_{\beta}f(t)=\left \{ \textstyle\begin{array}{l@{\quad}l} \frac{f(\beta(t))-f(t)}{\beta(t)-t},&{t}\neq{s_{0}},\\ {{f'}(s_{0})}, &{t}={s_{0}}, \end{array}\displaystyle \right . $$

where $f:I\rightarrow\mathbb{X}$ is a function defined on an interval $I\subseteq{\mathbb{R}}$, $\mathbb{X}$ is a Banach space and $\beta :I\rightarrow I$ is a strictly increasing continuous function defined on *I*, which has only one fixed point $s_{0}\in{I}$ and satisfies the inequality: $(t-s_{0})(\beta(t)-t)\leq0$ for all $t\in I$. The function *f* is said to be *β*-differentiable on *I*, if the ordinary derivative ${f'}$ exists at $s_{0}$. The *β*-difference operator yields the Hahn difference operator when $\beta(t)=qt+\omega$, $\omega>0$, $q \in(0,1)$, and the Jackson *q*-difference operator when $\beta(t)=qt$, $q \in(0,1)$; see [11–16]. In [10], [17, Chapter 2], the definition of the *β*-derivative, the *β*-integral, the fundamental theorem of *β*-calculus, the chain rule, Leibniz’s formula and the mean value theorem were introduced. In [18], the *β*-exponential, *β*-trigonometric and *β*-hyperbolic functions were presented. In [19], the existence and uniqueness of solutions of the *β*-initial value problem of the first order were established. In addition, an expansion form of the *β*-exponential function was deduced.

This paper is devoted for deducing some results of the solutions of the homogeneous second order linear *β*-difference equations which are based on $D_{\beta}$. In Section 2, we introduce the needed preliminaries of the *β*-calculus from [10, 17–19]. In Section 3, we prove the existence and uniqueness of solutions of the *β*-Cauchy problem of second order *β*-difference equations in a neighborhood of $s_{0}$. We also construct a fundamental set of solutions for the second order linear homogeneous *β*-difference equations when the coefficients are constants and study the different cases of the roots of their characteristic equations. Finally, we drive the Euler-Cauchy *β*-difference equation. Throughout this paper, *J* is a neighborhood of the unique fixed point $s_{0}$ of *β* and $\mathbb{X}$ is a Banach space. If *f* is *β*-differentiable two times over *I*, then the second order derivative of *f* is denoted by $D_{\beta}^{2}f=D_{\beta}(D_{\beta}f)$. Furthermore, $S(y_{0}, b)=\{y\in \mathbb{X}:\|y-y_{0}\|\leq b\}$ and the rectangle $R=\{(t,y)\in{{I}\times \mathbb{X}}:|t-s_{0}|\leq{a},\|y-y_{0}\|\leq{b}\}$, where *a*, *b* are fixed positive real numbers.

## Preliminaries

In this section, we present some needed results associated with the *β*-calculus from [10, 17–19].

### Lemma 2.1

*The following statements are true*:

(i) *The sequence of functions* $\{\beta^{k}(t)\}_{k=0}^{\infty}$ *converges uniformly to the constant function* $\hat{\beta}(t):=s_{0}$ *on every compact interval* $V \subseteq I$ *containing* $s_{0}$.
(ii) *The series* $\sum_{k=0}^{\infty}|\beta^{k}(t)-\beta^{k+1}(t)|$ *is uniformly convergent to* $|t-s_{0}| $ *on every compact interval* $V \subseteq I$ *containing* $s_{0}$.

### Lemma 2.2

*If* $f:I\rightarrow\mathbb{X}$ *is a continuous function at* $s_{0}$, *then the sequence* $\{f(\beta^{k}(t))\}_{k=0}^{\infty}$ *converges uniformly to* $f(s_{0})$ *on every compact interval* $V\subseteq I$ *containing* $s_{0}$.

### Theorem 2.3

*If* $f:I\rightarrow\mathbb{X}$ *is continuous at* $s_{0}$, *then the series* $\sum_{k=0}^{\infty}\| (\beta^{k}(t)-\beta^{k+1}(t) ) f(\beta ^{k}(t))\|$ *is uniformly convergent on every compact interval* $V \subseteq I$ *containing* $s_{0}$.

### Lemma 2.4

*Let* $f:{I}\rightarrow\mathbb{X}$ *be* *β*-*differentiable and* ${D}_{\beta}f(t)=0$ *for all* $t\in{I}$. *Then* $f(t)=f(s_{0})$ *for all* $t\in{I}$.

### Theorem 2.5

*Assume that* $f:{I}\rightarrow\mathbb {X}$ *and* $g:{I}\rightarrow\mathbb{R}$ *are* *β*-*differentiable functions on* *I*. *Then*:

(i) *the product* $fg:I\rightarrow\mathbb{X}$ *is* *β*-*differentiable on* *I* *and*
  $$\begin{aligned} {D}_{\beta}(fg) (t) &=\bigl({D}_{\beta}f(t) \bigr)g(t)+f\bigl(\beta(t)\bigr){D}_{\beta}g(t) \\ & =\bigl({D}_{\beta}f(t)\bigr)g\bigl(\beta(t)\bigr)+f(t){D}_{\beta}g(t), \end{aligned} $$
(ii) $f/g$ *is* *β*-*differentiable at* *t* *and*
  $${D}_{\beta} ({f}/{g} ) (t)=\frac{({D}_{\beta }f(t))g(t)-f(t){D}_{\beta}g(t)}{g(t)g(\beta(t))}, $$
  *provided that* $g(t)g(\beta(t))\neq{0}$.

### Theorem 2.6

*Assume* $f:{I}\to\mathbb{X}$ *is continuous at* $s_{0}$. *The function* *F* *defined by*

2.1 $$ F(t)=\sum_{k=0}^{\infty} \bigl( \beta^{k}(t)-\beta^{k+1}(t) \bigr)f\bigl(\beta^{k}(t) \bigr),\quad t\in{I} $$

*is a* *β*-*antiderivative of* *f* *with* $F(s_{0})=0$. *Conversely*, *a* *β*-*antiderivative* *F* *of* *f* *vanishing at* $s_{0}$ *is given by* (2.1).

### Definition 2.7

Let $f:{I}\rightarrow{\mathbb{X}}$ and $a,b\in{I}$. The *β*-integral of *f* from *a* to *b* is

$$\int^{b}_{a}f(t)\,d_{\beta}{t}= \int^{b}_{s_{0}}f(t)\,d_{\beta}{t}- \int ^{a}_{s_{0}}f(t)\,d_{\beta}{t}, $$

where

$$\int^{x}_{s_{0}}f(t)\,d_{\beta}{t}=\sum ^{\infty}_{k=0} \bigl(\beta^{k}(x)- \beta^{k+1}(x) \bigr)f\bigl(\beta^{k}(x)\bigr),\quad x\in{I}, $$

provided that the series converges at $x=a$ and $x=b$. *f* is called *β*-integrable on *I* if the series converges at *a* and *b* for all $a,b\in{I}$. Clearly, if *f* is continuous at $s_{0}\in{I}$, then *f* is *β*-integrable on *I*.

### Definition 2.8

The *β*-exponential functions $e_{p,\beta}(t)$ and $E_{p,\beta}(t)$ are defined by

2.2 $$\begin{aligned} e_{p,\beta}(t)=\frac{1}{\prod_{k=0}^{\infty }[1-p(\beta^{k} (t))(\beta^{k}(t)-\beta^{k+1}(t))]} \end{aligned}$$

and

2.3 $$\begin{aligned} E_{p,\beta}(t)=\prod_{k=0}^{\infty} \bigl[1+ p\bigl(\beta^{k}(t)\bigr) \bigl(\beta^{k} (t) - \beta^{k+1}(t) \bigr) \bigr], \end{aligned}$$

where $p:I \rightarrow\mathbb{C}$ is a continuous function at $s_{0}$ and both infinite products are convergent to a non-zero number for every $t\in I$ and $e_{p,\beta}(t)=\frac {1}{E_{p,\beta}(t)}$.

It is worth mentioning that both products in (2.2) and (2.3) are convergent since $\sum_{k=0}^{\infty} | p(\beta^{k}(t)) (\beta^{k}(t)-\beta ^{k+1}(t) ) |$ is uniformly convergent. See [18, Definition 2.1].

### Theorem 2.9

*The* *β*-*exponential functions* $e_{p,\beta}(t)$ *and* $E_{-p,\beta }(t)$ *are*, *respectively*, *the unique solutions of the* *β*-*initial value problems*:

$$\begin{gathered} D_{\beta}y(t)= p(t)y(t),\quad y(s_{0})=1, \\ D_{\beta}y(t)=-p(t)y\bigl(\beta(t)\bigr), \quad y(s_{0})=1.\end{gathered} $$

### Theorem 2.10

*Assume that* $p,q:I\rightarrow\mathbb {C}$ *are continuous functions at* $s_{0}\in I$. *The following properties are true*:

(i) $\frac{1}{e_{p,\beta}(t)}=e_{-p/[1+(\beta(t)-t)p]}(t)$,
(ii) $e_{p,\beta}(t)e_{q,\beta}(t)=e_{p+q+(\beta(t)-t)pq}(t)$,
(iii) $e_{p,\beta}(t)/e_{q,\beta}(t)=e_{(p-q)/[1+(\beta(t)-t)q]}(t)$.

### Definition 2.11

The *β*-trigonometric functions are defined by

$$\begin{gathered} \cos_{p,\beta}(t)=\frac{ e_{ip,\beta}(t)+e_{-ip,\beta }(t)}{2}, \\ \sin_{p,\beta}(t)=\frac{e_{ip,\beta} (t)-e_{-ip,\beta}(t)}{2i}. \end{gathered}$$

### Theorem 2.12

*For all* $t\in I$. *The following relation holds true*:

$$\begin{aligned} e_{ip,\beta}(t)=\cos_{p,\beta}(t)+i\sin_{p,\beta}(t). \end{aligned}$$

### Theorem 2.13

*Assume that the function* $f:R\rightarrow {\mathbb{X}}$ *is continuous at* $(s_{0},y_{0})\in{R}$ *and satisfies the Lipschtiz condition* (*with respect to* *y*)

$$ \big\| f(t,y_{1})-f(t,y_{2})\big\| \leq{L}\|y_{1}-y_{2} \|, \quad\textit{for all } (t,y_{1}), (t,y_{2})\in{R}. $$

*Then the* *β*-*initial value problem* $D_{\beta}{y(t)}=f(t,y)$, $y(s_{0})=y_{0}$, $t\in{I}$ *has a unique solution on* $[s_{0}-\delta,s_{0}+\delta]$, *where* *L* *is a positive constant and* $\delta=\min\{a,\frac{b}{Lb+M},\frac{\rho}{L}\}$ *with* $M=\sup_{(t,y)\in{R}}\|f(t,y)\|<\infty$, $\rho\in(0,1)$.

## Main results

In this section, we prove the existence and uniqueness of solutions of the *β*-Cauchy problem of second order *β*-difference equations in a neighborhood of $s_{0}$. Furthermore, we construct a fundamental set of solutions for the second order linear homogeneous *β*-difference equations when the coefficients are constants and study the different cases of the roots of their characteristic equations. Finally, we derive the Euler-Cauchy *β*-difference equation.

### Existence and uniqueness of solutions

#### Theorem 3.1

*Let* $f_{i}(t,y_{1},y_{2}):I \times\prod_{i=1}^{2} S_{i}(x_{i}, b_{i})\rightarrow{\mathbb{X}}$, $s_{0}\in I$, *such that the following conditions are satisfied*:

(i) *for* $y_{i}\in S_{i}(x_{i},b_{i})$, $i=1,2$, $f_{i}(t,y_{1},y_{2})$ *are continuous at* $t=s_{0}$,
(ii) *there is a positive constant* *A* *such that*, *for* $t\in I$, $y_{i}, \tilde{y}_{i}\in S_{i}(x_{i},b_{i})$, $i=1,2$, *the following Lipschitz condition is satisfied*:

$$\big\| f_{i}(t,y_{1},y_{2})-f_{i}(t, \tilde{y}_{1},\tilde{y}_{2})\big\| \leq A \sum _{i=1}^{2}\|y_{i}-\tilde{y}_{i} \|. $$

*Then there exists a unique solution of the* *β*-*initial value problem*, *β*-*IVP*,

3.1 $$ D_{\beta}y_{i}(t)=f_{i}\bigl(t,y_{1}(t),y_{2}(t) \bigr),\quad y_{i}(s_{0})=x_{i}\in {\mathbb{X}}, i =1,2, t \in I. $$

#### Proof

Let $y_{0}=(x_{1},x_{2})^{T}$ and $b=(b_{1},b_{2})^{T}$, where $(\cdot ,\cdot)^{T}$ stands for vector transpose. Define the function $f:I\times \prod_{i=1}^{2}S_{i}(x_{i},b_{i})\rightarrow{\mathbb{X}}\times{\mathbb{X}}$ by $f(t,y_{1},y_{2})= (f_{1}(t,y_{1},y_{2}),f_{2}(t, y_{1}, y_{2}) )^{T}$. It is easy to show that system (3.1) is equivalent to the *β*-IVP

3.2 $$ D_{\beta}y(t)=f\bigl(t,y(t)\bigr),\quad y(s_{0})=y_{0}. $$

Since each $f_{i}$ is continuous at $t=s_{0}$, *f* is continuous at $t=s_{0}$. The function *f* satisfies the Lipschitz condition because for $y,\tilde {y}\in\prod_{i=1}^{2}S_{i}(x_{i},b_{i})$,

$$\begin{aligned} \big\| f(t,y)-f(t,\tilde{y}) \big\| &= \big\| f(t,y_{1},y_{2})-f(t, \tilde{y}_{1}, \tilde{y}_{2}) \big\| \\ &=\sum_{i=1}^{2} \big\| f_{i}(t,y_{1},y_{2})-f_{i}(t, \tilde{y}_{1},\tilde {y}_{2},) \big\| \\ &\leq A\sum_{i=1}^{2}\|y_{i}- \tilde{y}_{i}\|= A\|y-\tilde{y}\|.\end{aligned} $$

Applying Theorem 2.13, see the proof in [19], there exists $\delta>0$ such that (3.2) has a unique solution on $[s_{0},s_{0}+\delta]$. Hence, the *β*-IVP (3.1) has a unique solution on $[s_{0},s_{0}+\delta]$. □

#### Corollary 3.2

*Let* $f(t,y_{1},y_{2})$ *be a function defined on* $I\times\prod_{i=1}^{2} S_{i}(x_{i},b_{i})$ *such that the following conditions are satisfied*:

(i) *for any values of* $y_{i}\in S_{i}(x_{i},b_{i})$, $i=1,2$, *f* *is continuous at* $t=s_{0}$,
(ii) *f* *satisfies the Lipschitz condition*
  $$\big\| f(t,y_{1},y_{2})-f(t,\tilde{y}_{1}, \tilde{y}_{2}) \big\| \leq A\sum_{i=1}^{2} \|y_{i} -\tilde{y}_{i}\|, $$
  *where* $A>0$, $y_{i},\tilde{y}_{i}\in S_{i}(x_{i},b_{i})$, $i=1,2$ *and* $t \in I$. *Then*
  3.3 $$\begin{aligned} D_{\beta}^{2}y(t)=f\bigl(t,y(t),D_{\beta}y(t)\bigr),\quad D_{\beta}^{i-1}y(s_{0})=x_{i}, i=1,2 \end{aligned}$$
  *has a unique solution on* $[s_{0},s_{0} +\delta]$.

#### Proof

Consider equation (3.3). It is equivalent to (3.1), where $\{\phi_{i}(t)\}_{i=1}^{2}$ is a solution of (3.1) if and only if $\phi_{1}(t)$ is a solution of (3.3). Here,

$$\begin{aligned} f_{i}(t,y_{1},y_{2})=\left \{ \textstyle\begin{array}{l@{\quad}l} y_{2},& i=1, \\ f (t,y_{1},y_{2}),& i=2. \end{array}\displaystyle \right . \end{aligned}$$

Hence, by Theorem 3.1, there exists $\delta>0$ such that system (3.1) has a unique solution on $[s_{0},s_{0}+\delta]$. □

The following corollary gives us the sufficient conditions for the existence and uniqueness of the solutions of the *β*-Cauchy problem (3.3).

#### Corollary 3.3

*Assume the functions* $a_{j}(t):I\rightarrow \mathbb{C}$, $j=0,1,2$, *and* $b(t):I\rightarrow{\mathbb{X}}$ *satisfy the following conditions*:

(i) $a_{j}(t), j=0,1,2$ *and* $b(t)$ *are continuous at* $s_{0}$ *with* $a_{0}(t)\neq0$ *for all* $t \in I$,
(ii) $a_{j}(t)/a_{0}(t)$ *is bounded on* *I*, $j=1,2$. *Then*
  3.4 $$ \begin{gathered} a_{0}(t)D_{\beta}^{2}y(t)+ a_{1}(t)D_{\beta}y(t)+a_{2}(t)y(t)=b(t), \\ D_{\beta}^{i-1}y(s_{0})= x_{i},\quad x_{i} \in{\mathbb{X}}, i=1,2, \end{gathered} $$
  *has a unique solution on subinterval* $J\subseteq I$, $s_{0}\in J$.

#### Proof

Dividing by $a_{0}(t)$, we get

3.5 $$ D_{\beta}^{2}y(t)=A_{1}(t)D_{\beta}y(t)+A_{2}(t)y(t)+B(t), $$

where $A_{j}(t)=-a_{j}(t)/a_{0}(t)$ and $B(t)=b(t)/a_{0}(t)$. Since $A_{j}(t)$ and $B(t)$ are continuous at $t=s_{0}$, the function $f(t,y_{1},y_{2})$, defined by

$$f(t,y_{1},y_{2})=A_{1}(t)y_{2}+A_{2}(t)y_{1}+B(t), $$

is continuous at $t=s_{0}$. Furthermore, $A_{j}(t)$ is bounded on *I*. Consequently, there is $A>0$ such that $|A_{j}(t)|\leq A$ for all $t \in I$. We can see that *f* satisfies the Lipschitz condition with Lipschitz constant *A*. Thus, $f(t,y_{1},y_{2})$ satisfies the conditions of Corollary 3.2. Hence, there exists a unique solution of (3.5) on *J*. □

### Fundamental solutions of linear homogeneous *β*-difference equations

The second order homogeneous linear *β*-difference equation has the form

3.6 $$ a_{0}(t)D_{\beta}^{2}y(t)+a_{1}(t)D_{\beta}y(t)+a_{2}(t)y(t)=0,\quad t \in I, $$

where the coefficients $a_{0}(t)\neq0$, $a_{j}(t)$, $j=1,2$ are assumed to satisfy the conditions of Corollary 3.3.

#### Lemma 3.4

*If the function* *y* *is a solution of the homogeneous equation* (3.6), *such that* $y(s_{0})=0$ *and* $D_{\beta}y(s_{0})=0$, $s_{0}\in I$, *then* $y(t)=0$, *for all* $t\in J$.

#### Proof

By Corollary 3.3, if $x_{i}=0$, $i=1,2$ in the *β*-IVP (3.4), which has a unique solution on *J*, then *y* such that $y(t)=0$ for all $t \in J$ is a unique solution of the *β*-difference equation (3.6), which satisfies the given initial conditions $y(s_{0})=0$, $D_{\beta}y(s_{0})= 0$. Hence we have the desired result. □

#### Theorem 3.5

*The linear combination* $c_{1}y_{1}+c_{2}y_{2}$ *of any two solutions* $y_{1}$ *and* $y_{2}$ *of the homogeneous linear* *β*-*difference equation* (3.6) *is also a solution of it in* *J*, *where* $c_{1}$ *and* $c_{2}$ *are arbitrary constants*.

#### Proof

The proof is straightforward. □

#### Theorem 3.6

*Let* $y_{1}$ *and* $y_{2}$ *be any two linearly independent solutions of the* *β*-*difference equation* (3.6) *in* *J*. *Then every solution* *y* *of* (3.6) *can be expressed as a linear combination* $y=c_{1}y_{1}+c_{2}y_{2}$.

#### Proof

Let

$$\phi=\left ( \textstyle\begin{array}{c} y\\ D_{\beta}y \end{array}\displaystyle \right ),\qquad \phi_{1}= \left ( \textstyle\begin{array}{c} y_{1}\\ D_{\beta}y_{1} \end{array}\displaystyle \right ),\qquad \phi_{2}=\left ( \textstyle\begin{array}{c} y_{2}\\ D_{\beta}y_{2} \end{array}\displaystyle \right ), $$

be the solutions of the linear system $D_{\beta}y_{i}(t)=a_{i}(t)y_{i}(t)$, $i=1,2$, corresponding, respectively, to the solutions $y_{1}$, $y_{2}$ of homogeneous linear *β*-difference equation (3.6). Since $y_{1},y_{2}$ are linearly independent in *J*, then $\phi_{1}$, $\phi_{2}$ are linearly independent in *J*. Then there exist two constants $c_{1}$, $c_{2}$ such that $\phi=c_{1}\phi _{1}+c_{2}\phi_{2}$. The first component of this is $y=c_{1}y_{1}+c_{2}y_{2}$. Thus the results hold. □

#### Definition 3.7

A set of two linearly independent solutions of the second order homogeneous linear *β*-difference equation (3.6) is called a fundamental set of it.

#### Theorem 3.8

*There exists a fundamental set of solutions of the second order homogeneous linear* *β*-*difference equation* (3.6).

#### Proof

By Corollary 3.3, there exist unique solutions $y_{1}$ and $y_{2}$ of equation (3.6), such that $y_{1}(s_{0})=1$, $D_{\beta}y_{1}(s_{0})=0$ and $y_{2}(s_{0})=0$, $D_{\beta}y_{2}(s_{0})=1$.

Suppose that $y_{1}$ and $y_{2}$ are linear dependent, so there exist constants $c_{1}$ and $c_{2}$ not both zero, such that

$$\begin{gathered} c_{1}y_{1}(t)+c_{2}y_{2}(t)= 0, \quad\text{for all } t\in J, \\ c_{1}D_{\beta}y_{1}(t)+c_{2}D_{\beta}y_{2}(t)= 0, \quad\text{for all } t\in J. \end{gathered}$$

We have $c_{1}=c_{2}=0$ at $t=s_{0}$, which is a contradiction. Thus the solutions $y_{1}$ and $y_{2}$ are linearly independent in *J*. Then there exists a fundamental set of the two solutions $y_{1}$ and $y_{2}$ of equation (3.6). □

#### Definition 3.9

Let $y_{1}$, $y_{2}$ be *β*-differentiable functions. Then we define the *β*-Wronskian of the functions $y_{1} $, $y_{2}$, defined on *I*, by

$$\begin{aligned} W_{\beta}(y_{1},y_{2}) (t)= \left \vert \textstyle\begin{array}{c@{\quad}c} y_{1}(t)& y_{2}(t)\\ D_{\beta}y_{1}(t)& D_{\beta}y_{2}(t) \end{array}\displaystyle \right \vert ,\quad t\in I. \end{aligned}$$

#### Lemma 3.10

*Let* $y_{1}(t)$, $y_{2}(t)$ *be functions defined on* *I*. *Then*, *for any* $t\in I$, $t \neq s_{0}$,

3.7 $$ D_{\beta}W_{\beta}(y_{1},y_{2}) (t)= \left \vert \textstyle\begin{array}{c@{\quad}c} y_{1}(\beta(t)) & y_{2}(\beta(t)) \\ D_{\beta}^{2}y_{1}(t) & D_{\beta}^{2}y_{2}(t). \end{array}\displaystyle \right \vert . $$

#### Proof

Since $W_{\beta}(y_{1},y_{2})(t)=y_{1}(t)D_{\beta}y_{2}(t)-y_{2}(t)D_{\beta}y_{1}(t)$, then

$$ D_{\beta}W_{\beta}(y_{1},y_{2}) (t)=y_{1}\bigl(\beta(t)\bigr)D_{\beta }^{2}y_{2}(t)-y_{2} \bigl(\beta(t)\bigr)D_{\beta}^{2}y_{1}(t), $$

which is the desired result. □

#### Theorem 3.11

*Assume that* $y_{1}(t)$ *and* $y_{2}(t)$ *are two solutions of equation* (3.6). *Then their* *β*-*Wronskian*, $W_{\beta}$,

$$W_{\beta}(y_{1},y_{2}) (t)=e_{-r_{1}(t)+r_{2}(t)(\beta(t)-t),\beta} W_{\beta}(y_{1},y_{2} ) (s_{0}),\quad t\in I , $$

*where* $r_{1}(t)=\frac{a_{1}(t)}{a_{0}(t)}$ *and* $r_{2}(t)=\frac{a_{2} (t)}{a_{0}(t)}$ *satisfy the conditions of Corollary* 3.3.

#### Proof

Since $y_{1}$ and $y_{2}$ are solutions of equation (3.6), from (3.7) we have

$$\begin{aligned} D_{\beta}W_{\beta}(y_{1},y_{2}) (t)&= \left \vert \textstyle\begin{array}{c@{\quad}c} y_{1}(\beta(t))&y_{2}(\beta(t)) \\ - \frac{a_{1}(t)}{a_{0}(t)} D_{\beta}y_{1}(t)&- \frac{a_{1} (t)}{a_{0}(t)} D_{\beta}y_{2}(t) \end{array}\displaystyle \right \vert + \left \vert \textstyle\begin{array}{c@{\quad}c} y_{1}(\beta(t))&y_{2}(\beta(t)) \\ - \frac{a_{2} (t)}{a_{0}(t)}y_{1}(t) &-\frac{a_{2}(t)}{a_{0}(t)}y_{2}(t) \end{array}\displaystyle \right \vert \\ &= - \frac{a_{1}(t)}{a_{0}(t)} \left \vert \textstyle\begin{array}{c@{\quad}c} y_{1}(t)&y_{2}(t) \\ D_{\beta}y_{1} (t) &D_{\beta}y_{2}(t) \end{array}\displaystyle \right \vert + \frac{a_{2}(t)}{a_{0}(t)}\bigl(\beta(t)-t\bigr) \left \vert \textstyle\begin{array}{c@{\quad}c} y_{1}(t)&y_{2}(t) \\ D_{\beta}y_{1}(t) &D_{\beta}y_{2}(t) \end{array}\displaystyle \right \vert \\ &= \bigl[-r_{1}(t)+r_{2}(t) \bigl(\beta(t)-t\bigr) \bigr]W_{\beta}(y_{1},y_{2}) (t),\end{aligned} $$

which has the solution

$$ W_{\beta}(y_{1},y_{2}) (t)= W_{\beta}(y_{1},y_{2}) (s_{0})e_{-r_{1}(t)+r_{2}(t)(\beta(t)-t),\beta} ,\quad t \in I. $$

 □

Using Theorem 3.11 and Lemma 3.4, we can prove the following corollaries.

#### Corollary 3.12

*Two solutions* $y_{1}$ *and* $y_{2}$ *of* *β*-*difference equation* (3.6) *are linearly dependent in* *J* *if and only if* $W_{\beta}(y_{1},y_{2})(t)=0$, *for all* $t\in J$.

#### Corollary 3.13

*The value of* $W_{\beta}(y_{1},y_{2})(t)$ *of* *β*-*difference equation* (3.6) *either is zero or unequal to zero for all* $t \in J$.

### Homogeneous equations with constant coefficients

Equation (3.6) can be written as

3.8 $$ Ly(t)=aD_{\beta}^{2}y(t)+bD_{\beta}y(t)+cy(t)=0, $$

where *a*, *b*, and *c* are constants. The characteristic polynomial of equation (3.8) is

3.9 $$ P(\lambda)=a\lambda^{2}+b \lambda+c=0, $$

where $y(t)=e_{\lambda,\beta}(t)$ is a solution of equation (3.8). Since equation (3.9) is a quadratic equation with real coefficients, it has two roots, which may be real and different, real but repeated, or complex conjugates.

*Case 1: real and different roots of the characteristic equation* (*3.9*)*.*

Let $\lambda_{1}$ and $\lambda_{2}$ be real roots with $\lambda_{1}\neq \lambda_{2}$, then $y_{1}(t)=e_{\lambda_{1},\beta}(t)$ and $y_{2}(t)=e_{\lambda_{2},\beta }(t)$ are two solutions of equation (3.8). Therefore,

$$ y(t)=c_{1}e_{\lambda_{1},\beta}(t)+c_{2}e_{\lambda_{2},\beta}(t) $$

is a general solution of equation (3.8), with

$$ c_{1}=\frac{D_{\beta}y_{0}-y_{0}\lambda_{2}}{\lambda_{1}-\lambda_{2}}e_{-\lambda _{1},\beta}(s_{0})\quad \text{and}\quad c_{2}=\frac{y_{0}\lambda_{1}-D_{\beta}y_{0}}{\lambda _{1}-\lambda_{2}} e_{-\lambda_{2},\beta}(s_{0}). $$

#### Example 3.14

Find the solution of the *β*-initial value problem

$$\begin{aligned} D_{\beta}^{2}y(t)+5D_{\beta}y(t)+6y(t)=0,\quad y(s_{0})=2, D_{\beta}y(s_{0})=3. \end{aligned}$$

By assuming that $y(t)=e_{\lambda,\beta}(t)$, we obtain the solution

$$y(t)=9e_{-2,\beta}(t)-7e_{-3,\beta}(t). $$

*Case 2: complex roots of the characteristic equation* (*3.9*).

Let $\lambda_{1}=\nu+i\mu$ and $\lambda_{2}=\nu-i\mu$, where *ν* and *μ* are real numbers. Then $y_{1}(t)=e_{(\nu+i\mu),\beta}(t)$ and $y_{2}(t)=e_{(\nu-i\mu),\beta}(t)$ are two solutions of equation (3.8). By Theorems 2.10, 2.12, $e_{(\nu+i\mu),\beta }(t)=e_{\nu,\beta}(t) e_{\frac{i\mu}{1+\nu(\beta(t)-t)},\beta}(t)$. So,

$$ e_{(\nu+i\mu),\beta}(t)=e_{\nu,\beta}(t) \bigl(\cos _{\frac{\mu}{1+\nu(\beta(t)-t)},\beta}(t)+i \sin_{\frac{\mu}{1+\nu(\beta (t)-t)},\beta}(t) \bigr). $$

We have

$$y_{1}(t)+y_{2}(t)=2 e_{\nu,\beta}(t) \cos_{\frac{\mu}{1+\nu (\beta(t)-t)},\beta}(t) $$

and

$$y_{1}(t)-y_{2}(t)=2i e_{\nu,\beta}(t) \sin_{\frac{\mu}{1+\nu(\beta (t)-t)},\beta}(t). $$

Therefore,

$$u(t)=e_{\nu,\beta}(t)\cos_{\frac{\mu}{1+\nu(\beta(t)-t)},\beta}(t) \quad\text{and} \quad v(t)= e_{\nu,\beta}(t)\sin_{\frac{\mu}{1+\nu(\beta(t)-t)},\beta} (t) $$

are two solutions of equation (3.8). If the *β*-Wronskian of *u* and *v* is not zero, then *u* and *v* form a fundamental set of solutions. The general solution of equation (3.8) is

$$y(t)=c_{1}e_{\nu,\beta}(t)\cos_{\frac{\mu}{1+\nu(\beta(t)-t)},\beta }(t)+c_{2}e_{\nu,\beta}(t) \sin_{\frac{\mu}{1+\nu(\beta(t)-t)},\beta}(t), $$

where $c_{1}$ and $c_{2}$ are arbitrary constants.

#### Example 3.15

Find the general solution of

3.10 $$ D_{\beta}^{2}y(t)+D_{\beta}y(t)+y(t)=0. $$

The characteristic equation is $\lambda^{2}+\lambda+1=0$, and its roots are

$$\lambda_{1,2}=\frac{-1}{2}\pm i\frac{\sqrt{3}}{2}. $$

Thus, the general solution of equation (3.10) is

$$y(t)=c_{1} e_{-1/2,\beta}(t)\cos_{\frac{\sqrt{3}/2}{1-1/2(\beta(t)-t)},\beta }(t)+c_{2}e_{-1/2,\beta}(t) \sin_{\frac{\sqrt{3}/2}{1-1/2(\beta(t)-t)},\beta}(t). $$

*Case 3: repeated roots.*

Consider the case that the two roots $\lambda_{1}$ and $\lambda_{2}$ are equal, so

$$\lambda_{1}=\lambda_{2}=-b/2a. $$

Therefore, the solution $y_{1}(t)=e_{-b/2a,\beta} (t)$ is one solution of the *β*-difference equation (3.8), and we give the second solution by the following example:

#### Example 3.16

Solve the *β*-difference equation

3.11 $$ D_{\beta}^{2}y(t)+4D_{\beta}y(t)+4y(t)=0. $$

The characteristic equation is $(\lambda+2)^{2}=0$, so $\lambda_{1}=\lambda _{2}=-2$. Therefore, $y_{1}(t)= e_{-2,\beta}(t)$ is a solution of equation (3.11). To find the second solution, let $y(t)=v(t)e_{-2,\beta}(t)$. Then ${D_{\beta}^{2}v(t)=0}$. Therefore, $v(t)=c_{1}t+c_{2}$, where $c_{1}$ and $c_{2}$ are arbitrary constants. Then the general solution is

$$ y(t)=c_{1}te_{-2,\beta}(t)+c_{2}e_{-2,\beta}(t), $$

where the two solutions $y_{1}(t)=e_{-2,\beta}(t)$ and $y_{2}(t)=te_{-2,\beta}(t)$ form a fundamental set of solutions of equation (3.11).

### Euler-Cauchy *β*-difference equation

The Euler-Cauchy *β*-difference equation takes the form

3.12 $$ t\beta(t)D_{\beta}^{2}y(t)+atD_{\beta}y(t)+by(t)=0,\quad t\in I, t\neq s_{0}, $$

where *a*, *b* are constants. The characteristic equation of (3.12) is given by

3.13 $$ \lambda^{2}+(a-1)\lambda+b=0. $$

#### Theorem 3.17

*If the characteristic equation* (3.13) *has two distinct roots* $\lambda_{1}$ *and* $\lambda_{2} $, *then a fundamental set of solutions of* (3.12) *is given by* $e_{\lambda_{1}/t,\beta}(t)$ *and* $e_{\lambda _{2}/t,\beta}(t)$.

#### Proof

Let $y(t)=e_{\lambda/t,\beta}(t)$, where *λ* is a root of equation (3.13). It follows that

$$D_{\beta}y(t)=\frac{\lambda}{t}y(t),\qquad D_{\beta}^{2}y(t)= \frac{\lambda ^{2}-\lambda}{t\beta(t)}y(t). $$

Consequently, we have

$$t\beta(t)D_{\beta}^{2}y(t)+atD_{\beta}y(t)+by(t)=\bigl( \lambda^{2}+(a-1)\lambda +b\bigr)y(t)=0. $$

Assume that $\lambda_{1}$ and $\lambda_{2}$ are distinct roots of the characteristic equation (3.13). Then, we have

$$\lambda_{1}+\lambda_{2}=1-a,\qquad \lambda_{1} \lambda_{2}=b. $$

Moreover, $W_{\beta}(e_{\lambda_{1}/t,\beta},e_{\lambda_{2}/t,\beta})(t)\neq 0$, since $\lambda_{1}\neq\lambda_{2}$. Hence, $e_{\lambda_{1}/t,\beta}(t)$ and $e_{\lambda_{2}/t,\beta}(t)$ form a fundamental set of solutions of (3.12). □

The following theorem gives us the general solution of the Euler-Cauchy *β*-difference equation in the double root case.

#### Theorem 3.18

*Assume that* $1/\beta(t)$ *is bounded on* *I* *and* $0\notin I$. *Then the general solution of the Euler*-*Cauchy* *β*-*difference equation*

3.14 $$ t\beta(t)D_{\beta}^{2}y(t)+(1-2\gamma)tD_{\beta}y(t)+ \gamma^{2}y(t)=0,\quad t\in I, $$

*is given by*

$$y(t)=c_{1}e_{\frac{\gamma}{t},\beta}(t)+c_{2} e_{\frac {\gamma}{t},\beta}(t) \int_{s_{0}}^{t}\frac{ e_{\frac{-1}{\beta(\tau )},\beta}}{1+\frac{\gamma}{\tau}(\beta(\tau)-\tau)}\,d_{\beta}\tau. $$

#### Proof

The characteristic equation of (3.14) is

$$\lambda^{2}-2\gamma\lambda+\gamma^{2}=0. $$

Then the characteristic roots are $\lambda_{1}=\lambda_{2}=\gamma$. Hence one linearly independent solution of equation (3.14) is $y_{1}(t)=e_{\frac{\gamma}{t},\beta}(t)$. To obtain the second linearly independent solution, we can rewrite equation (3.14) in the form

3.15 $$ D_{\beta}^{2}y(t)+r_{1}(t)D_{\beta}y(t)+r_{2}(t)y(t)=0, $$

where $r_{1}(t)=\frac{1-2\gamma}{\beta(t)}$ and $r_{2}(t)=\frac{\gamma ^{2}}{t\beta(t)}$. Consequently,

$$-r_{1}(t)+r_{2}(t) \bigl(\beta(t)-t\bigr)= \frac{\gamma^{2}}{t}-\frac{(\gamma-1)^{2}}{\beta(t)}. $$

Let *u* be a solution of equation (3.15) such that $u(s_{0})=0$, $D_{\beta}u(s_{0})=1$. Then

$$W_{\beta}( e_{\frac{\gamma}{t},\beta},u) (t)=e_{-r_{1}(t)+r_{2}(t)(\beta (t)-t),\beta}(t)= e_{\frac{\gamma^{2}}{t}-\frac{(\gamma-1)^{2}}{\beta (t)},\beta}(t). $$

By Theorem 2.5, we find that *u* satisfies the following *β*-difference equation:

$$\begin{aligned} D_{\beta}\biggl(\frac{u}{e_{\frac{\gamma}{t},\beta}}\biggr) (t)&=\frac{W_{\beta}( e_{\frac{\gamma}{t},\beta},u)(t)}{ e_{\frac{\gamma}{t},\beta}(t)e_{\frac {\gamma}{\beta(t)},\beta}(\beta(t))} \\ &=\frac{ e_{\frac{\gamma^{2}}{t}-\frac{(\gamma-1)^{2}}{\beta(t)},\beta}(t)}{ e_{\frac{\gamma}{t},\beta}^{2}(t)(1+\frac{\gamma}{t}(\beta(t)-t))}.\end{aligned} $$

Then

$$u(t)=e_{\frac{\gamma}{t}}(t) \int_{s_{0}}^{t}\frac{ e_{\frac {\alpha^{2}}{\tau}-\frac{(\gamma-1)^{2}}{\beta(\tau)},\beta}(\tau)}{ e_{\frac{\gamma}{\tau},\beta}^{2}(\tau)(1+\frac{\gamma}{\tau}(\beta (\tau)-\tau))}\,d_{\beta}\tau. $$

Also,

$$\frac{e_{\frac{\gamma^{2}}{t}-\frac{(\gamma-1)^{2}}{\beta (t)},\beta}(t)}{ e_{\frac{\gamma}{t},\beta}^{2}(t)}=e_{\frac{-1}{\beta (t)},\beta}(t). $$

Therefore,

$$y(t)=c_{1} e_{\frac{\gamma}{t},\beta}(t)+c_{2}e_{\frac {\gamma}{t},\beta}(t) \int_{s_{0}}^{t}\frac{e_{\frac{-1}{\beta(\tau)},\beta}(\tau)}{1+\frac {\gamma}{\tau}(\beta(\tau)-\tau)}\,d_{\beta}\tau $$

is the general solution of equation (3.14). □

## Conclusion

In this paper, the existence and uniqueness of solutions of the *β*-Cauchy problem of second order *β*-difference equations were proved. Moreover, a fundamental set of solutions for second order linear homogeneous *β*-difference equations when the coefficients are constants was constructed. Also, the different cases of the roots of the characteristic equations of these equations were studied. Finally, the Euler-Cauchy *β*-difference equation was derived.
